# A Connection Method between Ultrahard PtW8 Wire and a Au Thick Film Based on Parallel-Gap Resistance Microwelding

**DOI:** 10.3390/ma13132911

**Published:** 2020-06-29

**Authors:** Mingqiang Pan, Minghui Tu, Jizhu Liu

**Affiliations:** 1 School of Mechanical and Electric Engineering, Soochow University, Suzhou 215123, China; pmqwl@126.com; 2 Jiangsu Provincial Key Laboratory of Advanced Robotics, Soochow University, Suzhou 215123, China; 3 Robotics and Microsystems Center, Soochow University, Suzhou 215123, China

**Keywords:** ultrahard PtW8 wire, Au thick film, parallel-gap welding, thermal gas sensor, joint formation

## Abstract

To meet the application requirements of a thermal gas sensor, it is necessary to realize a bond connection between PtW8 wire with a Au thick film. However, the physical properties, such as the melting point and hardness, of the two materials differ greatly. In this study, the parallel-gap resistance microwelding was introduced into the bonding connection between PtW8 wire and a Au thick film in the thermal gas sensor. The feasibility of the method was analyzed theoretically and the experimental system was established and studied. A scanning electron microscope (SEM) was used to analyze the morphology of the cross-section of the welded joint. The results showed that there was no obvious transition layer at the interface region but there were relatively dense welds. At the same time, it was found that the melted Au wetted and climbed on the surface of the platinum-tungsten alloy, which may have been the key to forming the joint. Elements were observed to have a spatial distribution gradient within the cross-section of the welding line, revealing that mutual diffusion occurred in the parallel-gap resistance microwelding, where this diffusion behavior may be the basic condition for forming the joint. Finally, the influence of the welding voltage, time, and force on the joint strength was also studied, where the joint strength could be up to 5 cN.

## 1. Introduction

In recent years, gas sensors have been widely used in industrial production and people’s daily lives [1,2,3]. Due to people’s increasing awareness of their safety and environmental protection, researchers and developers pay increasingly more attention to the development of gas sensors. Among a wide variety of gas sensors, thermal gas sensors have the advantages of a simple structure, high sensitivity, excellent selectivity, all solid-state, good reliability, etc., and have been a research hotspot in the sensor field [4,5].

Electrode leads are essential components for signal transmission and the support of sensitive bodies in thermal gas sensors [6,7]. The support of the electrode lead affects the performance of the thermal gas sensor. Feng et al. proposed four Pt wires with a certain stiffness as the electrode leads of the gas sensor, and the gold slurry was dropped on the Pt wire and the Au electrode, which were then subjected to a high-temperature sintering process to form the joint [8,9]. However, the Pt wire underwent a high-temperature sintering process (700 °C for 1 h), which caused the Pt wire to be fully annealed, affecting the quality of the gas sensor. For a Pt wire diameter of less than 50 μm, low strength was displayed and breakage occurred. Lee et al. used 12 Pt wires with a certain rigidity as the electrode leads of the catalytic combustion gas sensor to support sensitive components, micro-hot plates, and other components while reducing the heat loss of the heating plate [10]. In recent years, to expand the application of sensors, researchers have studied the interconnection of leads and pads. For example: Pt wire/Pt thin film [11,12,13,14], Pd wire/Pt thin film [12,15], Pt wire/PtIr pad [16], etc. However, the physical properties of the materials studied are not much different and the joint formation during the bonding process is not clear. In research on the resistance microwelding of crossed Pt-Ir alloy wire and 316 LVM stainless steel wires, Huang et al. showed that increasing the wetting of molten metal on the surface of the joint can significantly improve the performance of the joint [17,18,19].

With the rapid development of technology, higher requirements are placed on the high-temperature resistance and support of electrode leads; to this end, platinum-tungsten alloys have become a new application direction. PtW8 wire has higher strength, stiffness, and high-temperature resistance than Pt wire, and is the most promising electrode material, as shown in Table 1 [15]. To expand the application requirements of thermal gas sensors, the PtW8 wire should be bonded onto the Au thick film. However, the melting point, hardness, and stiffness of platinum-tungsten alloys are large, making it difficult to bond. Meanwhile, the mechanism of the joint formation of soft and hard materials is not clear.

**Figure 1:**
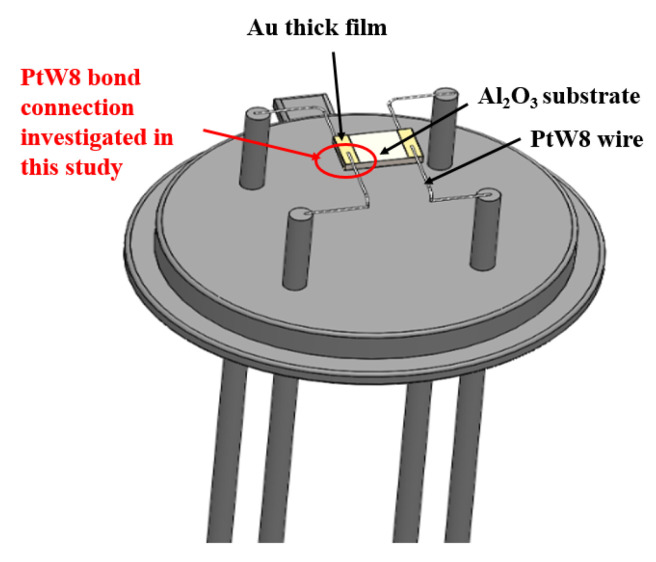
Schematic diagram of a typical planar parathermal gas sensor.

For this reason, this study introduced parallel-gap resistance microwelding into the bonding connection between ultrahard PtW8 wire and a Au thick film in a thermal gas sensor. A typical planar parathermal gas sensor is shown in Figure 1 [20]. The feasibility of the method was analyzed theoretically and an experimental system was built and studied. This method not only has the advantages of strong controllability and a simple process but can also reduce the difficulty in making the thermal gas sensor.

## 2. Process Design and Experiment

### 2.1. Process Design of Parallel-Gap Resistance Microwelding

Resistance microwelding is a fusion-welding process [21]. Two characteristic steps are required for resistance microwelding: (1) apply a certain pressure to the electrode such that the workpiece is in close contact, and (2) pass a current through the workpiece using resistance heat to melt the base metal and form a nugget. Since it is influenced by many factors, resistance microwelding of PtW8 and Au is a complicated problem. First, as mentioned earlier, the physical properties of PtW8 and Au have great differences. Second, the size effects become stronger with the decrease of the size of the PtW8 wire and the Au thick film. All the above factors make resistance microwelding of PtW8 and Au difficult to conduct. Therefore, a suitable method is needed to overcome the above disadvantages.

**Figure 2:**
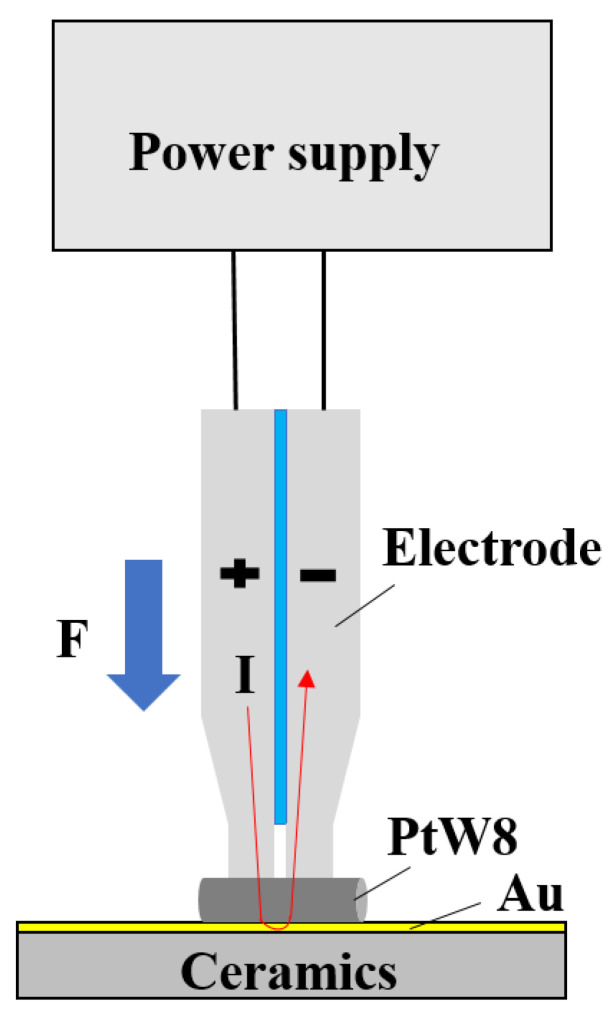
Schematic diagram for parallel-gap resistance microwelding.

The basic concept of parallel-gap resistance microwelding of PtW8 wire and a Au thick film is shown in Figure 2. The experimental setup mainly consists of a power supply, an electrode, and a workpiece. The power supply is the core component of parallel-gap resistance microwelding equipment [22]. The performance of the power supply directly affects the precision of the welding current and time control. The capacitor-stored pulse power supply can obtain a large instantaneous current and is less difficult to control. The electrode plays an important role in pressing the workpiece during the welding process, generating an average heat distribution, providing a large current to the welding site, and promoting the cooling of the welded parts [23]. The use of molybdenum electrodes can reduce other problems, such as electrode bonding, and the bonding quality has been significantly improved [24]. For platinum-tungsten alloys with a high melting point and high-hardness wire, an electrode made of molybdenum can be selected. The electrode provides additional heat to soften the wire to improve the bonding quality. Second, the gap and width of the electrode can affect the current density and thus the quality of the bonding [24]. When reducing the electrode gap and width, it is also necessary to consider the difficulty of the manufacturing process to better design the structure of the electrode and improve the quality of bonding.

**Figure 3:**
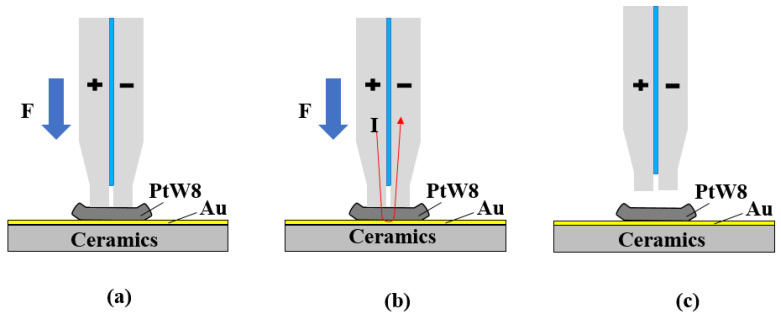
Schematic of the parallel-gap resistance microwelding process: (**a**) preloading stage, (**b**) welding stage, and (**c**) after welding.

A schematic diagram of a parallel-gap resistance microwelding process is shown in Figure 3. At the beginning of the process, the electrode applies pressure through the driving mechanism, pressing the ultrahard PtW8 wire tightly on the Au thick film, as shown in Figure 3a. During the preloading stage, the contaminants on the surface are crushed and the materials reach intimate contact. The power supply then releases a large current for a certain period, as shown in Figure 3b. At the beginning of the welding stage, the current flows from the positive electrode to the negative electrode through the PtW8 and the Au thick film. Due to the resistance heat caused by the current, partial melting takes place between the PtW8 and the Au thick film, where a preliminary bond is formed at this time. As the welding stage progresses, due to the difference in the physical properties of the two materials, the Au melts but the platinum-tungsten alloy does not melt. At this time, liquid Au spreads on the surface of the platinum-tungsten alloy to form a joint. After the welding is completed, the electrode is separated from the workpiece, as shown in Figure 3c.

### 2.2. Experiment

Platinum-tungsten alloy wires and Au-plated ceramic substrates were used in this study. The diameter of each platinum-tungsten alloy wire was 40 μm. The size of the Al_2_O_3_ substrate was 1 × 1.5 × 0.25 mm, and the Al_2_O_3_ substrate was coated with a 10 μm thick Au film. The material properties of PtW8 and Au are given in Table 2. The performance characteristics of PtW8 differ greatly from those of Au in many aspects, as shown in Table 2. For example, the melting temperature of PtW8 is about 800 °C higher than that of Au, the electrical resistivity is about 31 times that of Au, and the thermal conductivity of Au is about 4 times that of PtW8. Because of the differences in performance characteristics between PtW8 wire and Au thick films, resistance microwelding (RMW) is difficult to achieve. Before welding, all materials were ultrasonically cleaned in acetone for 10 min.

A self-developed parallel-gap microresistance welding machine was used in the experiment. The adjustable voltage range was 0–2.8 V, the electrode force adjustment range was 0–4000 g, and the output pulse time range adjustment range was 0–99.9 ms. The electrode material was molybdenum. The cross-section size of the electrode connector was 250 μm × 250 μm. The welding parameter group was set as follows: the voltage range was 0.8–1.1 V, the electrode force range was 100–600 g, and the output pulse time range was 0–90 ms.

To facilitate the observation and analysis of the morphology and composition of the cross-section of the welded joint after parallel-gap resistance microwelding, cold inlay technology was used to prepare the sample. The cold inlay technology was utilized as follows: (1) The welded sample was clamped and fixed using a sample holder and then placed vertically in the center of the circular mold. The sample holder was required to ensure that the welded sample was perpendicular to the bottom of the mold. (2) The curing agent and resin were mixed at a mass ratio of 1:3, then stirred evenly and poured into the mold to wait for its solidification. (3) Sandpaper with grades 800#, 1000#, 2000#, 3000#, and 5000# were used to grind the sample. During the grinding process, the sample was continuously observed under the microscope until it was ground to the area near the welded spot. (4) A diamond polishing agent with a particle size of 0.5 μm was used to perform polishing on the inlay samples. During the polishing process, the sample was observed under the microscope until the welded spot was clearly visible. Finally, the polished surface was cleaned and dried with anhydrous ethanol and a hair dryer to remove stains and polishing powder.

**Figure 4:**
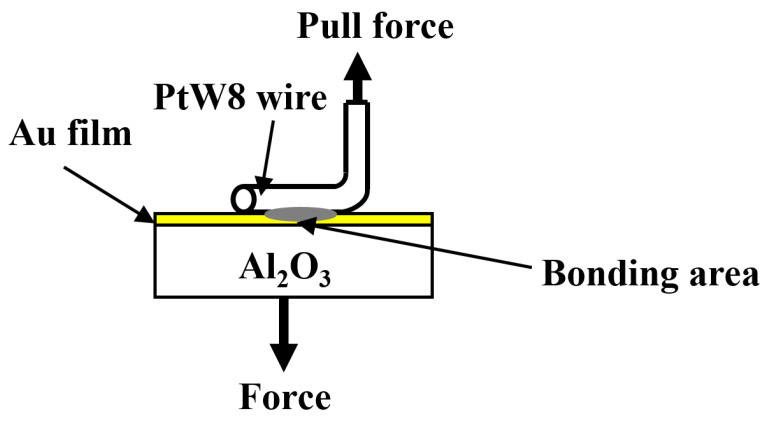
Schematic of the tensile test.

The joint quality was evaluated using a tensile test, as shown in Figure 4. During the test, the force was continuously increased until failure occurred. The maximum force before joint failure was used as the test result. The joint surface morphology, as well as the cross-sectional morphology and composition, were examined using a EVO18 scanning electron microscope (SEM, Carl Zeiss AG, Jena, Germany) with an energy-dispersive X-ray spectroscopy (EDS) is equipped.

## 3. Results and Discussion

### 3.1. Welded Joint and Microstructure

**Figure 5:**
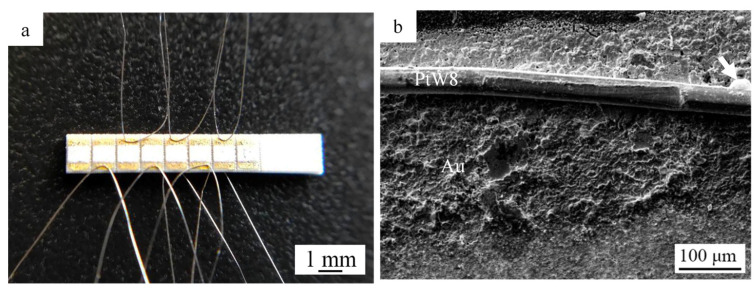
(**a**) Welded sample of a PtW8 wire and a Au thick film. (**b**) Surface morphology of a welded joint.

The experimental results show that the system and method used successfully welded the PtW8 wire with a diameter of 40 μm to the Au thick film. The physical figure of the parallel-gap resistance microwelding sample and the surface morphology of the welded joint under a scanning electron microscope are given in Figure 5a,b, respectively. The corresponding parallel-gap resistance microwelding parameters were a welding voltage of 0.95 V, a welding time of 80 ms, and a welding force of 200 g. As can be seen from Figure 5a, the welded sample had a good consistency. As can be seen from Figure 5b, the PtW8 wire had fewer shape variables and the bonding interface was relatively flat. At the same time, the significant melting of materials that had been squeezed out of the interfacial zone, primarily the Au, was observed, as shown in Figure 5b. However, under this condition, the molten Au could not wet the surface of the PtW8 wire well, as evidenced by the formation of large solidified balls around the PtW8 wire (as indicated by arrows in Figure 5b). The above phenomenon can be explained as follows: the physical parameters, such as the melting point and hardness of PtW8 and Au are quite different, and the temperature was lower due to insufficient heat input and hence a poor wettability.

**Figure 6:**
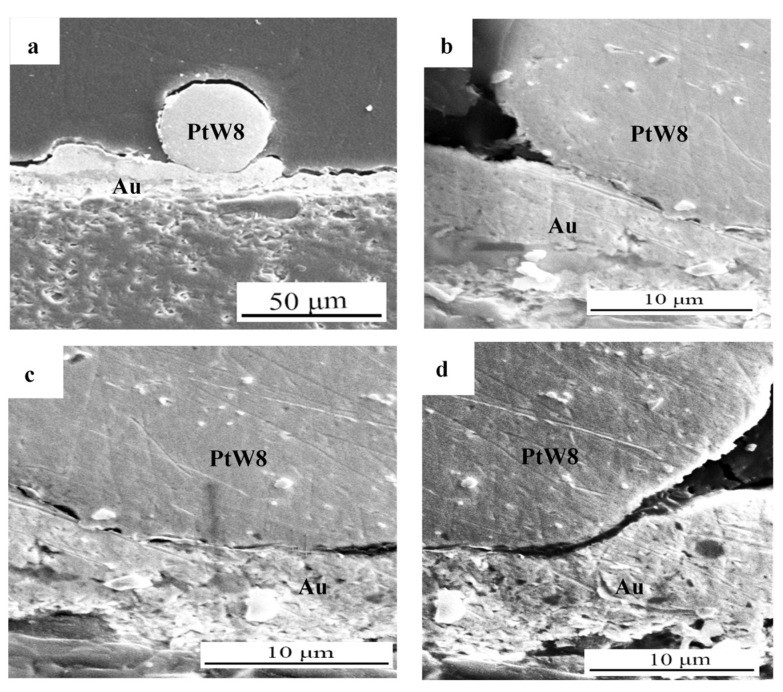
(**a**) SEM cross-sectional images of joints, (**b**) enlarged view on the left side of the cross-section, (**c**) middle enlarged view of cross-section, and (**d**) enlarged view on the right side of the cross-section.

SEM low-magnification and high-magnification cross-sectional images at the bond interface of the joints made using 0.95 V, 80 ms, and 200 g are illustrated in Figure 6. It can be observed from Figure 6a that the PtW8 did not deform, whereas the upper surface of the Au thick film appeared to be bent and the bonded area between PtW8 and Au presented an arc. This phenomenon may be caused by the difference in material properties. Compared with PtW8, Au has a low melting point and hardness; therefore, plastic deformation or melting occurs first. Figure 6b–d shows a partial magnification of the bonded interface. The length of the fully bonded region was around 20 μm, as shown in Figure 6b,c. The unbonded area can be clearly seen in Figure 6d. This phenomenon may have been caused by the uneven distribution of forces, resulting in the differential distribution of heat.

**Figure 7:**
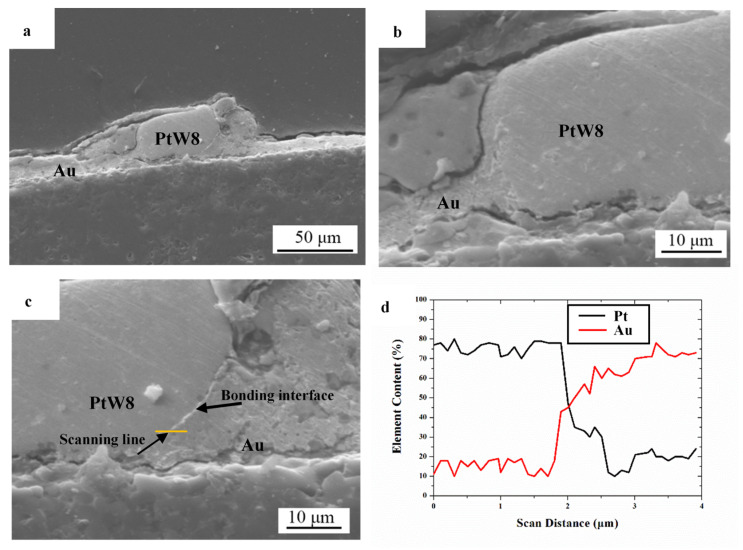
(**a**) SEM cross-sectional images of joints, (**b**) enlarged view on the left side of the cross-section, (**c**) enlarged view on the right side of the cross-section, and (**d**) chemical elements analysis of the cross-section of the bonding interface.

A welding pressure of 500 g, a welding voltage of 1.1 V, and a welding time of 16 ms created the joint conditions that made the cross-section shown in the scanning electron microscopy (SEM) diagram in Figure 7. Figure 7a–c shows that under high heat and a high welding force, the platinum-tungsten alloy softened and deformed, and the liquid Au showed good wetting, spreading, and climbing phenomena on the surface of Pt-W alloy. The increase of heat energy resulted in a higher temperature, a decreased contact angle, the same length of time under the condition of an applied force, PtW8 softening deformation, and liquid wetting spreading and climbing of the Au. Cracks were also observed at the intermediate bonded interface, as shown in Figure 7b,d. This phenomenon may have been due to excessive heat energy and force, resulting in cracks at the bonded interface.

The element distribution within the cross-section of the bonding interface was obtained via line analysis with EDS, as shown in Figure 7d. It can be seen that the constituent elements of Pt and Au presented a significant concentration gradient along the longitudinal direction in the bonded region, indicating that mutual diffusion of materials occurred in the process of the parallel-gap welding. When the current flowed through the material, the resistance heat generated by the resistance made the temperature of the welding area rise rapidly and the transient high-temperature environment made the materials partially melt. At the same time, under the action of the force, the liquid Au wetted and spread to the PtW8 surface. The instantaneous high-temperature environment promoted the mutual diffusion of elements. We believe that such wetting spread and mutual diffusion are the basic conditions for forming good joints in PtW8 and Au parallel-gap welding.

### 3.2. Preloading Analysis of Parallel-Gap Resistance Microwelding

**Figure 8:**
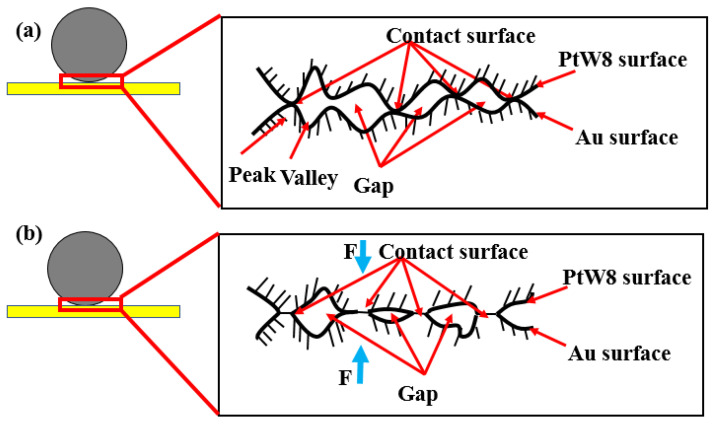
Influence of the force on the bonding interface: (**a**) not pressurized and (**b**) pressurized.

In the process of parallel-gap resistance microwelding, the welding pressure directly affected the initial contact area, and then the interface contact resistance. The change of interface contact resistance affected the distribution of the temperature field in the welding process and affected the joint quality directly [25,26]. The schematic diagram of the impact of the welding pressure on the bonding interface is shown in Figure 8. As can be seen from Figure 8b, when the welding force was applied to the workpiece, the contact bump increased and the contact point underwent elastic deformation. The contact changed from the peak-to-peak point contact to surface contact and the contact area increased. Contact resistance can be expressed as:

(1) $$R_{c}=\rho \frac{D}{WL},$$

where  ρ is the interface resistivity, *D* is the gap, *W* is the width of the section, and *L* is the axial length.

According to Equation (1), with the increase of welding pressure, the gap decreases, the surface roughness decreases, the contact area increases, and the contact resistance shows a decreasing trend. When the welding pressure is too low, the contact resistance is large. According to Joule’s law, the heat generated by the current will also increase rapidly, and the surface of the joint is prone to spatter and defects of electrode bonding, resulting in poor quality. Because the thickness of the welding pad coating is usually only a few microns, when the welding force is too large, it is likely to produce a stratification phenomenon or even cause mechanical damage to the welding pad, where the stratification and damage of the coating will become a hidden danger of the solder joint reliability such that the failure mode from the solder joint failure causes failure of the coating.

**Figure 9:**
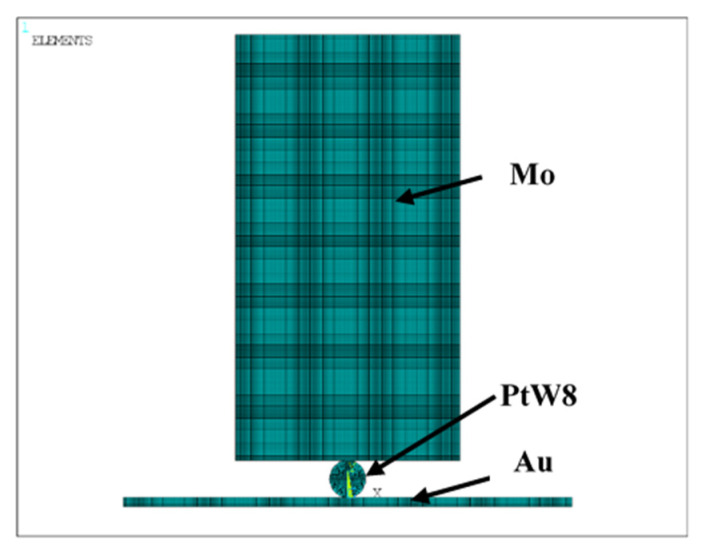
Finite element model of the preloading process.

To better understand the contact behavior in the preloading stage, and because the preloading process is difficult to be measured experimentally, this study used software ANSYS15.0 to analyze the preloading process. To improve the simulation efficiency, only the electrode, PtW8, and Au were modeled, as shown in Figure 9.

**Figure 10:**
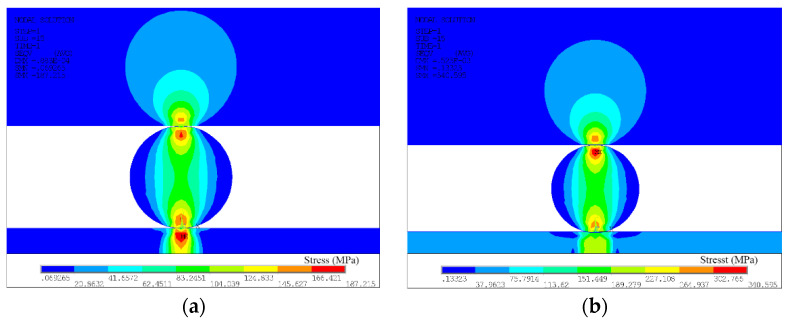
Stress distribution of the von Mises stress: (**a**) F = 0.2 N and (**b**) F = 0.5 N.

The stress distribution diagram in the preloading stage is shown in Figure 10. Figure 10a shows the stress distribution diagram between the workpiece and between the workpiece and the electrode under the welding pressure F = 0.2 N. It can be clearly seen from the figure that the stress distribution presented a symmetrical distribution along the centerline of the *Y*-axis. The maximum stress occurred below the central position of the Au thick film, and the contact center between the PtW8 and Mo electrode was inclined to the PtW8, with a value of about 187.215 Mpa; both of which failed to reach the yield strength of the material. Figure 10b shows the stress distribution diagram between the workpiece and between the workpiece and the electrode under the condition of welding pressure F = 0.5 N. It can be observed from the figure that the Au layer produced plastic deformation and part of the platinum-tungsten alloy was pressed into the Au thick film. The contact form between the Au and the PtW8 changed from point contact to line contact, and the stress distribution changes. For the PtW8 and the Mo electrode, the maximum stress still occurred below the contact point of the PtW8, where the maximum stress was about 340.596 MPa.

### 3.3. The Temperature Field of the Parallel-Gap Resistance Microwelding

In the process of the PtW8 and Au parallel-gap resistance microwelding, the heat was mainly provided by resistance heat. The equation for resistance heat is as follows:

(2) $$Q=I^{2}Rt,$$

where *I* represents the welding current, *t* represents the welding time, and *R* represents the total resistance. The total resistance *R* was mainly composed of the intrinsic resistance of the Mo electrode, PtW8, and Au film, as well as the contact resistance of the contact site. The self-resistance of the Mo electrode, PtW8, and Au film was mainly related to the inherent properties of the conductor, such as the structure and resistivity. In the process of RMW, the contact resistance plays a decisive role in the whole process [27,28]. The contact resistance can be expressed by Equation (1).

**Figure 11:**
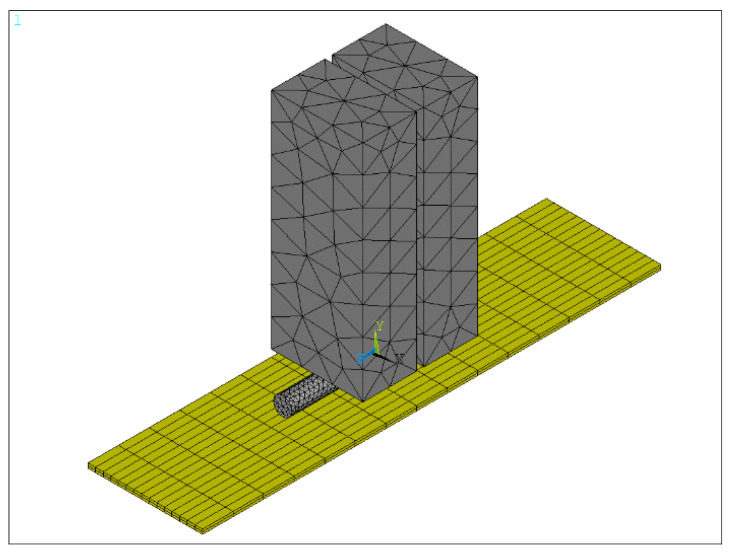
Finite element model of the welding process.

Based on the results of the preload analysis, we reconfigured the model at the power-on stage to improve the accuracy of the analysis. To provide temperature field analysis of the Au thick film and the bonding mechanism of the joint, the finite element method was used to simulate the welding process between the PtW8 wire and Au thick film. To improve the simulation efficiency and consider the accuracy of the temperature distribution, the direct coupling method was used to conduct the three-dimensional modeling of the Mo electrode, Au thick film, and PtW8 wire, and the model was simplified appropriately. The finite element model of the welding stage is shown in Figure 11.

**Figure 12:**
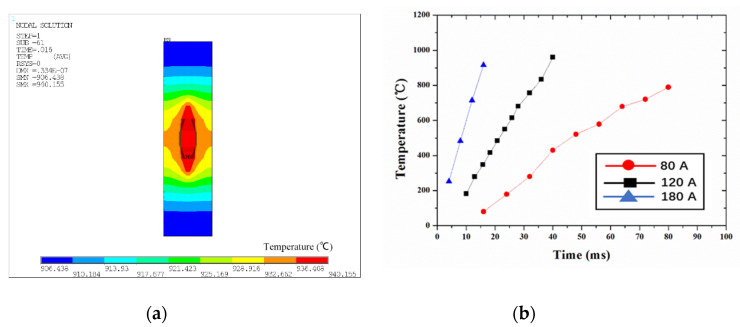
(**a**) The temperature field distribution of the Au thick film. (**b**) The time variation of the maximum temperature of the Au thick film using different currents.

The temperature field distribution of the Au thick film and the time variation of the maximum temperature of the Au thick film under different current conditions are shown in Figure 12. Figure 12a shows the temperature distribution of the Au thick film when the welding current was 180 A and the welding time was 16 ms. It can be clearly seen that the maximum temperature distribution was elliptical and the maximum temperature was about 940 °C, which is close to the melting point of Au. Since the contact area is not an ideal plane, local melting will occur under this condition. At this temperature, the diffusion between atoms can be accelerated. It can be seen from Figure 12b that both the current and time influenced the temperature, where the current had the greatest influence.

### 3.4. Wetting Behavior of Liquid Au on the Surface of the Platinum-Tungsten Alloy Wire

The wetting behavior of liquid Au on the platinum-tungsten alloy is one of the important factors affecting the formation of the joint. The wettability of solid platinum-tungsten alloy by liquid Au can be qualitatively determined using Young’s equation [29]. For spreading wetting, the size of the indirect antenna between liquid Au and a solid platinum-tungsten alloy directly reflects the wetting situation, while the size of the contact angle depends on the balance of interfacial tension at the tri-junction point at the spreading front [30]. According to Young’s equation (Equation (3)), the contact angle of the beam, which measures the degree of wettability, can be calculated as follows:

(3) $$cos\alpha =\frac{\sigma_{sg}-\sigma_{sl}}{\sigma_{lg}},$$

where cosα is the wetting coefficient, $\sigma_{sg}$ is the solid–gas surface tension, $\sigma_{sl}$ is the solid–liquid surface tension, and $\sigma_{lg}$ is liquid–gas surface tension.

According to Equation (3), the smaller the contact angle, the better the spreading effect. According to Young’s equation, increasing $\sigma_{sg}$ or decreasing $\sigma_{sl}$ and/or $\sigma_{lg}$ can promote wetting. From the perspective of thermodynamics, the interfacial tension is related to the specific surface enthalpy, which is related to the physical properties, composition, and temperature of each phase; therefore, the wetting angle must be affected by these factors.

Surface tension affects the wetting behavior of liquid Au. The relationship between the liquid Au surface tension (mN·m^−1^) and temperature (°C) is expressed in Equation (4) [31]:

(4) $$\sigma_{Au}=1162-0.18\left(T-1065\right)\;(T>1065\;℃).$$

The surface tension of liquid Au decreases with the increase of temperature within a certain temperature range. PtW8 is an inert metal, and the temperature coefficient of its solid–gas surface tension is very small, which does not change with the temperature. The temperature coefficient of the liquid–solid interface is between these two. Therefore, according to Equation (3), the contact angle tends to decrease. Temperature affects not only the contact angle but also the climbing height of liquid Au on the surface of the platinum-tungsten alloy. The wetting height *H* can be expressed as:

(5) $$H=\frac{2\sigma_{lg}\left(1-sin\alpha \right)}{\rho g},$$

where $\sigma_{lg}$ is the surface tension of liquid Au, ρ is the density of liquid Au, and g is gravity.

According to Equation (5), the wetting height is mainly related to the contact angle of liquid Au on the surface of the platinum-tungsten alloy. When the temperature rises, the contact angle becomes smaller, and Equation (5) shows that the wetting height increases.

**Figure 13:**
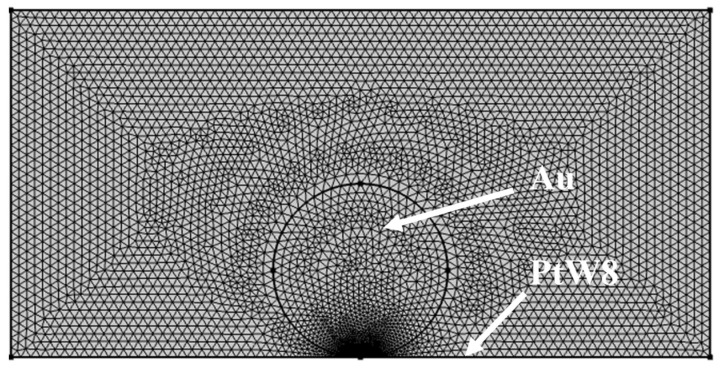
Finite element model of the spreading process.

The wetting spread of liquid Au on the surface of platinum-tungsten alloy is a prerequisite for forming a smooth surface and good performance joints. To analyze the wetting spread of liquid Au on the surface of platinum-tungsten alloy, a COMSOL Multiphysic two-phase flow level set was used in this study. To improve the simulation’s efficiency, a two-dimensional model was adopted. Its finite element model is shown in Figure 13.

**Figure 14:**
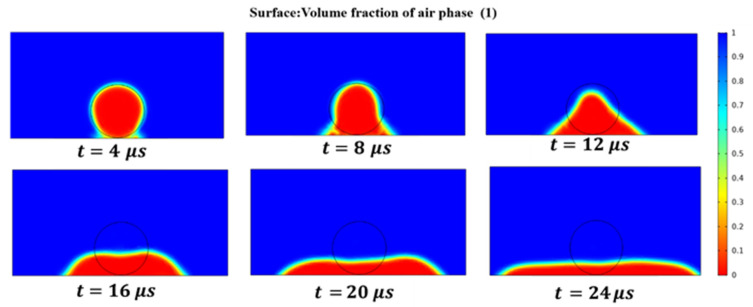
The spread of liquid Au on the surface of the platinum-tungsten alloy.

The spreading process of liquid Au on the surface of the platinum-tungsten alloy is shown in Figure 14. The blue area represents the area that is air, the red area represents the area that is liquid Au, the bottom is the platinum-tungsten alloy surface. As can be clearly seen from Figure 14, under the effect of gravity and surface tension, liquid Au rapidly spread out on the surface of the platinum-tungsten alloy in a very short time, and then reached the equilibrium state. In a time of *t* = 4–24 μs, liquid Au spread symmetrically outward at the three-phase interface as time went by, and its height decreased in the process of spreading. It can also be seen from Figure 14 that liquid Au had a better spreading effect on the surface of the platinum-tungsten alloy, which may be attributed to the material properties of platinum-tungsten alloy, which was conducive to the wetting and spreading of liquid Au on the surface of the platinum-tungsten alloy.

### 3.5. Bonding Mechanism

**Figure 15:**
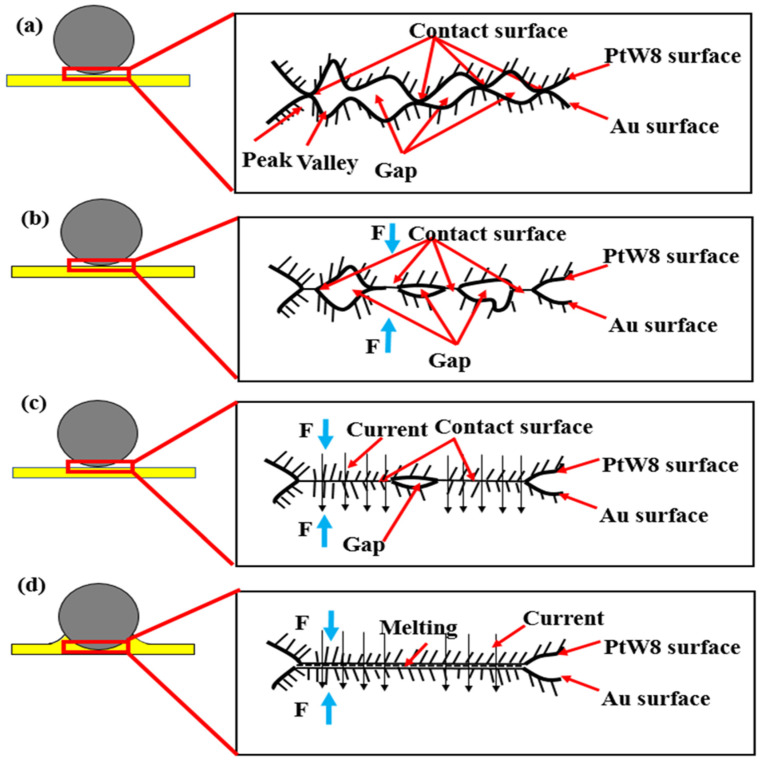
Schematic diagram of joint formation: (**a**) initial state, (**b**) deformation after pressing, (**c**) partial melting, and (**d**) molten metal wetting and spreading.

According to the surface morphology, cross-sectional morphology, and theoretical analysis of the joint, we can summarize the formation process of the joint, as shown in Figure 15. Before welding, the wire and board were stacked together. Due to the surface roughness of the material, the contact between the materials was not an ideal smooth plane but the point-to-point contact was made of countless peaks and peaks or peaks and valleys, as shown in Figure 15a. At the beginning of the experiment, the electrode applied a welding pressure through the driving mechanism to press the ultrahard PtW8 wire tightly on the Au thick film. Under the welding pressure, the contaminants on the surface of the material were crushed, and due to the difference in the physical properties of the two materials, the metal with a low strength began to deform. The number of contact points increased, and elastic deformation occurred at the contact points. The contact changed from point contact to surface contact, as shown in Figure 15b. The current was then applied for a certain period. The change of bonding interface under the power-on condition is shown in Figure 15c,d. When current flowed through the contact area of the PtW8 wire and Au thick film, the actual contact area between the PtW8 wire and the Au thick film was much smaller than the contact area due to the roughness of the contact area. Therefore, the current density at the contact region was very high and the contact volume was quite small compared with the material itself. These gave rise to a local increase in temperature at the contact region and the contact point was welded, provided that the temperature at the contact exceeded the melting point of the material, as shown in Figure 15c. As the current increased, due to the difference in the physical properties of the two materials, molten Au appeared. When the current continued to increase, increasingly more molten Au was created and the liquid Au began to wet, spread, and climb rapidly on the surface of the platinum-tungsten alloy, as shown in Figure 15d. Finally, a joint was formed. Through the above analysis, it can be concluded that the bonding mechanism of the parallel-gap resistance microwelding of PtW8 and Au was solid-state bonding. Under the action of a mechanical force, the oxides and pollutants were broken, and then under the action of thermal–force coupling, the oxides and pollutants were decomposed by heat, creating a relatively clean surface, wjere the mutual diffusion of atoms between the interface and the molten Au on the platinum-tungsten alloy allowed for surface wetting spread and climbing, thus forming the solid-state joint.

### 3.6. Factors Affecting Welded Quality

**Figure 16:**
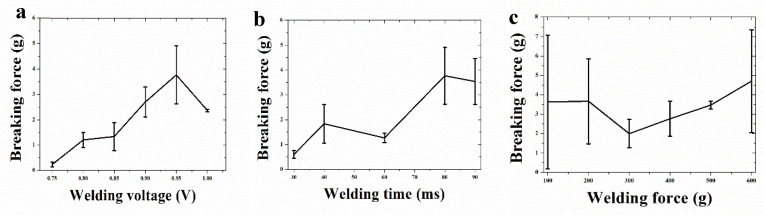
Factors affecting the welding quality: (**a**) welding voltage, (**b**) welding time, and (**c**) welding force.

The heat energy can be calculated as follows (Equation (6)):

(6) $$Q=\int_{0}^{t}\frac{U_{0}e^{-\frac{t}{RC}}}{R}dt,$$

where *R* is the total resistance, *C* is capacitance, *U*_0_ is the initial voltage, and *t* is the welding time. According to Equation (6), it can be known that voltage and time are proportional to the heat energy, and the welding voltage and time largely determine the amount of welding heat. Figure 16a shows the breaking force test results of the welded samples under different welding voltages when the welding time was 80 ms and the welding force was 200 g. As can be seen from Figure 16a, the breaking force gradually increased with the increase of the welding voltage in the range of 0.75–0.95 V, and the maximum value was about 4 g. When in the range of 0.95 V to 1.0 V, the fracture force decreased. If the voltage continues to increase, serious damage to Au and cracks in the ceramic substrate may occur. Therefore, the appropriate voltage setting should be selected to improve the breaking force and reduce the damage of the welded joints and Au thick film.

Figure 16 shows the breaking force test results of the welded samples for different welding times under the conditions of a welding voltage of 0.95 V and a welding force of 200 g. As can be seen from Figure 16b, in the welding time range of 30–80 ms, the welding quality improved with the increase of the welding time. This was due to the increase of the welding interface energy, which increased the contact area of the bonding interface and reduced the connection gap and voids. When the holding time was greater 80–90 ms, the connection quality was basically unchanged, and the breaking force was about 4 g at this time. A reasonable explanation for this behavior may be that the weld formation occurred in a relatively short period. In other words, in an optimal welding parameter setting, a peak temperature is reached quickly. The long holding time makes the welding heat concentrate too much on the electrode and less on the welding interface. It can be concluded that the welding time need not be maintained for a long time to reduce the influence of excess heat on the joint morphology and the worn electrode head.

Figure 16c shows the breaking force test results of the welded sample under different welding pressures at a welding voltage of 0.95 V and a welding time of 80 ms. Under the condition of a higher welding pressure, the deformation of the wire and the contact area of the weld was increased, resulting in greater bonding strength. However, the overall breaking force was small, mainly due to the higher melting point and hardness of PtW8; the overall deformation was small; the welding interface area was small; and low joint strength was displayed.

## 4. Conclusions

The purpose of this study was to investigate the PtW8 wire and Au thick-film weldability using parallel-gap resistance microwelding. Based on the experimental and theoretical analysis results, the major conclusions can be summarized as follows:

1. The ultrahard PtW8 wire was successfully bonded with the Au thick film using parallel-gap resistance microwelding under appropriate welding parameters. The joint strength could be up to 5 cN.
2. It was found that the parallel-gap resistance microwelding of PtW8 wire and Au thick film included the following stages: (1) deformation after pressing, (2) partial melting, (3) molten metal wetting and spreading, and (4) solid-state bonding.
3. Sufficient local heat generation was the key to high-quality welds because first, it generated sufficient molten metal, and second, it created plastic deformation, and third, it facilitated the wetting and spreading of the molten metal to expand the bonded area.
4. Optimizing only the welding voltage, time, and force was insufficient to achieve acceptable joint strength.

## Figures and Tables

**Table 1:** Material properties of Pt and PtW8.

Material	Melting Point (°C)	Elasticity Modulus (GPa)
Pt	1769	171
PtW8	1870	230

**Table 2:** Material properties.

Material	Melting Temperature (°C)	Electrical Resistivity (10^−8^ Ω∙m)	Thermal Conductivity (W∙m^−1^·K^−1^)	Elasticity Modulus (GPa)
PtW8	1870	62	71	230
Au	1064	2.21	318	79
